# Synthetic Receptors for Early Detection and Treatment of Cancer

**DOI:** 10.3390/bios13110953

**Published:** 2023-10-25

**Authors:** Frank Davis, Séamus P. J. Higson

**Affiliations:** Department of Chemical Engineering and Biotechnology, University of Cambridge, Philippa Fawcett Drive, Cambridge CB3 0AS, UK; fd417@cam.ac.uk

**Keywords:** cancer, early diagnosis of cancer, synthetic receptors for early detection of cancer, synthetic receptors for treatment of cancer, calixarene, cyclodextrin, cucurbituril

## Abstract

Over recent decades, synthetic macrocyclic compounds have attracted interest from the scientific community due to their ability to selectively and reversibly form complexes with a huge variety of guest moieties. These molecules have been studied within a wide range of sensing and other fields. Within this review, we will give an overview of the most common synthetic macrocyclic compounds including cyclodextrins, calixarenes, calixresorcinarenes, pillarenes and cucurbiturils. These species all display the ability to form a wide range of complexes. This makes these compounds suitable in the field of cancer detection since they can bind to either cancer cell surfaces or indeed to marker compounds for a wide variety of cancers. The formation of such complexes allows sensitive and selective detection and quantification of such guests. Many of these compounds also show potential for the detection and encapsulation of environmental carcinogens. Furthermore, many anti-cancer drugs, although effective in in vitro tests, are not suitable for use directly for cancer treatment due to low solubility, inherent instability in in vivo environments or an inability to be adsorbed by or transported to the required sites for treatment. The reversible encapsulation of these species in a macrocyclic compound can greatly improve their solubility, stability and transport to required sites where they can be released for maximum therapeutic effect. Within this review, we intend to present the use of these species both in cancer sensing and treatment. The various macrocyclic compound families will be described, along with brief descriptions of their synthesis and properties, with an outline of their use in cancer detection and usage as therapeutic agents. Their use in the sensing of environmental carcinogens as well as their potential utilisation in the clean-up of some of these species will also be discussed.

## 1. Introduction

Cancer is one of the world’s most serious diseases, leading to chronic illness for millions of people worldwide every year and in 2020 more than 10 million deaths [1]. Mitigating this situation requires both early detection of cancer before it is too late for treatment and after detection through, for example, a combination of surgical, radiological and chemotherapy procedures to cure, treat or manage the condition. Detection and determination of cancer is the first crucial step for curing any patient. A wide number of methods have been used to detect cancer; some of these involve physical-based techniques such as MRI and other scanning methods, while others rely on the selective determination of marker compounds produced by the cancer. Examples of such techniques include prostate-specific antigen, high levels of which can indicate prostate cancer and CA125—a marker protein for ovarian cancer. Typical detection methods include ELISA assays, which use antibodies as selective detection agents for these marker compounds. However, an alternative approach involves the development of artificial, non-biological systems for cancer detection, including such materials as aptamers. Other work, discussed within this review, focuses on the development of smaller macrocyclic compounds capable of being used within cancer-detecting approaches.

Chemotherapy is one of the major tools for mitigating and curing cancer. However, there are several major issues that can affect this approach. Many potential drug candidates prove to be too insoluble for use in cancer treatment and need to be solubilised. Unfortunately, many drug molecules are not selective in their action, being taken up by both healthy and cancerous cells [2]; this lack of specificity gives rise to the many side effects often associated with chemotherapy such as hair loss and damage to bone marrow and the intestinal tract [3,4]. There is therefore great interest in developing suitable moieties, which could act as smart delivery agents, solubilising the drug, aiding its selective transport to the cancer cells and then releasing it to serve its purpose. Such systems would reduce the collateral damage to the patient’s healthy cells while allowing for more effective destruction of the cancer.

Over recent decades, there has been a great deal of interest in the synthesis of a wide variety of macrocyclic compounds, which have the potential for selective binding to various targets and also the ability to encapsulate, solubilise and transport a wide range of compounds [5]. These compounds display several properties, making them especially suitable for these applications. Their synthesis can be controlled to allow the construction of systems with tailored binding properties. Also, for therapeutic applications, it is essential that the species used should either be stable under physiological conditions until they are excreted or, if they do break down, no harmful products are generated. Many of the macrocycles described in this work are highly stable or in the case of the cyclodextrins made up of harmless sugar units. Within this review, we will discuss several of these families of compounds such as calixarenes and the related calixresorcinarenes and pillarenes as well as cyclodextrins and cucurbiturils. The potential of these families of compounds for the sensing of cancer markers and carcinogens, binding to cancer cells and potential to act as anti-cancer drugs will be discussed. 

## 2. Calixarenes, Resorcinarenes and Pillarenes

**Figure 1:**
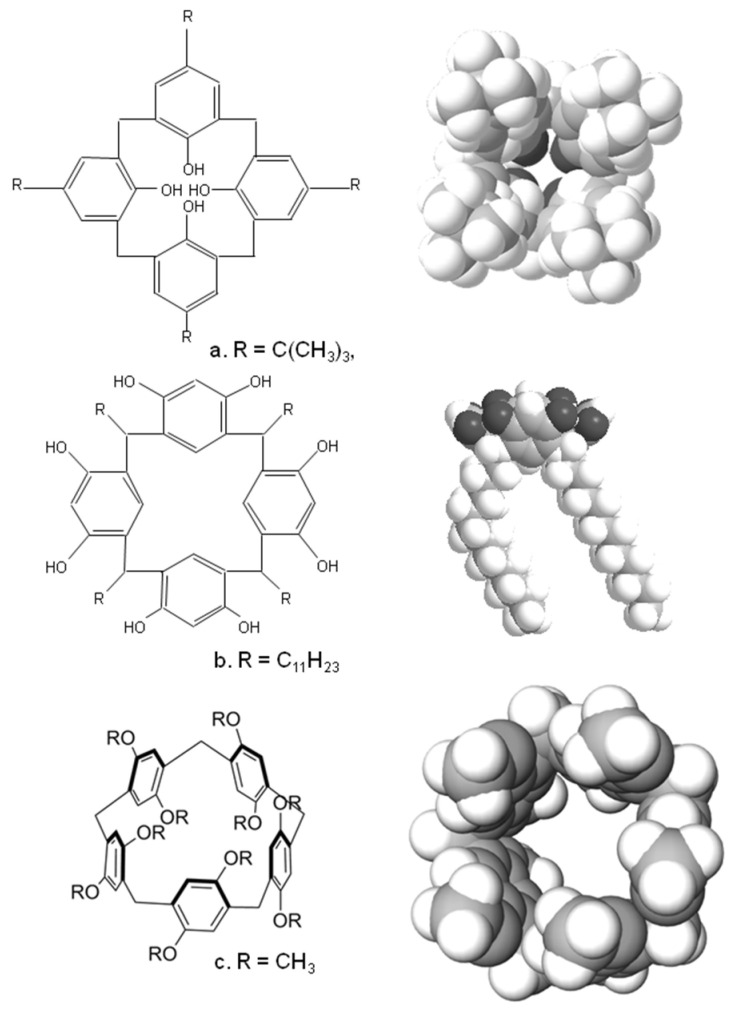
Typical structures and crystal structure of (**a**) a calix(4)arene (**b**) a calix(4)resorcinarene and (**c**) a pillar(5)arene.

Calixarenes are cyclic oligomers formed by acid or base-catalysed condensation or substituted phenols with formaldehyde [5,6]. Figure 1 shows the schematic structure and 3D structure of one of the simplest and earliest calixarenes formed by the condensation of *t*-butyl phenol with formaldehyde. As can be seen, there is a clear bowl-shaped structure, which led to their naming since they resemble a Greek bowl or “calix” [6]. There is a wide range of these molecules, since the substituents on the hydroxyl unit, the aromatic ring and the linking group can all be varied. The most common ring sizes for these oligomers are 4, 5, 6 or 8 benzene units in the macrocyclic ring, although much larger systems have been successfully synthesised [6,7]. Such molecules can exist in a wide variety of conformations; in Figure 1, we show the calix in its cone conformation—but other conformations do exist, namely, the partial cone, 1,2-alternate and 1,3-alternate [5,6,7]. Larger calixarenes have a much wider range of potential conformations. 

A similar family of compounds is the calixresorcinarenes, Figure 1b, which are synthesised by condensing aldehydes with resorcinol or substituted resorcinols such as pyrogallol [5,6]. These also have a bowl-shaped cavity ringed by hydroxyl groups; Figure 1b shows the crystal structure of a tetra-undecyl calix(4)resorcinarene formed by the acid-catalysed condensation of resorcinol with dodecanal [8]. Again, the hydroxyl groups, the 2-position on the benzene ring and the sidechains can all be modified; the ring sizes of the compounds are mainly tetramers, although a wide range of other ring sizes have been synthesised [5].

Finally, there are the pillarenes, which rather than resembling a bowl-shaped conformation, display a more tubular structure. These can be synthesised using a variety of substituted hydroquinone derivatives, which are condensed with formaldehyde. Cyclic pentamers and hexamers are the most common, and once again a wide variety of substituted pillarenes have been synthesised. Besides these species, a whole range of other macrocyclic species have been synthesised with a wide range of structures, many of which are reviewed here [9].

### Sensing Applications

Calixarenes have been shown to be suitable for the detection of biomarkers for cancer; for example, a water-soluble guanidinium substituted calix(5)arene was shown to be capable of selectively binding the phospholipid lysophosphatidic acid (LPA)—a biomarker shown to be useful for the early detection of ovarian cancers [10]. Combined with a fluorescent displacement assay, this protocol allowed quantification of LPA in mouse serum between 0 and 50 μmol L^−1^ with a limit of detection of 1.7 μmol L^−1^. The same calix also proved capable of binding trimethylamine N-oxide, a marker for medical conditions including colorectal cancer. Detection of trimethylamine N-oxide was possible with a linear range from 0 to 1.22 mmol L^−1^ and a limit of detection of 28.9 μmol L^−1^ [11]. Sarcosine is an amino acid, the presence of which in urine is a potential biomarker for prostate cancer. Carbon nanotubes could be modified with a phosphonate calix(4)resorcinarene and have been used to form a selective conductimetric sensor for sarcosine in the presence of other amino-acids with detection levels as low as 0.02 mmol L^−1^ [12].

Calixarenes could also be utilised as modification agents for gold nanoparticles and then further modified with peptides to develop sensing moieties for the human cancer biomarker oncoprotein Mdm2, with a limit of detection of 30 nmol L^−1^ [13]. An increased level of sarcosine and its derivatives in urine has been linked to the presence of prostate cancer. The use of a tetraphosphonate substituted for calix(4)resorcinarene combined with an electroluminescence protocol allowed for the determination of sarcosine in the μmol L^−1^ to mmol L^−1^ level, which covers the range between healthy and cancerous patients [14]. The use of calixarenes and other cavitands for sensing biological targets has also been reviewed [15]. 

As an alternative to testing blood or urine samples, a calix(4)arene that selectively binds to cancer cells has been developed. Cancer cells often have high levels of folate receptors, and therefore a calix(4)arene bearing four folate units and four 7-nitro-benzofurazan fluorophores [16] was synthesised. This molecule bound very strongly to HeLa cancer cells, allowing their accumulation of the calixarene intracellularly into the endo-lysosomal system and confocal fluorescence microscopy imaging of the cells. A water-soluble para-sulphonated calix(8)arene was also shown to strongly bind folic acid units and, when this calix was immobilised onto a black phosporene/polydopamine surface, allowed for the construction of a selective electrochemical sensor for folic acid units [17]. This sensor proved capable of determining levels of LNCaP cancer cells with a linear range of 2 × 10^2^–1 × 10^5^ cells mL^−1^ and a detection limit of 36 cells mL^−1^.

Once detection of cancer has been attained, the next step is to attempt a cure. Calixarenes and their derivatives have been used in studies that either directly use the calixarene as an anti-cancer agent or as a carrier for an anti-cancer drug moiety [7,18,19,20,21]. A calix(4)arene substituted with dimethylamino groups—OTX008 has been shown to inhibit Galectin-1, a carbohydrate-binding protein that has been implicated in cancer cell proliferation, invasion and tumour angiogenesis [22]. The use of OTX008 in micromolar concentrations was shown in vitro to inhibit the growth and proliferation of a number of human cancer cell lines. Other work used calix(4)arene aminophosphonates to inhibit the enzyme glutathione-S-transferase, an enzyme known to contribute to the development of resistance of cancer cells to drugs [23].

**Figure 2:**
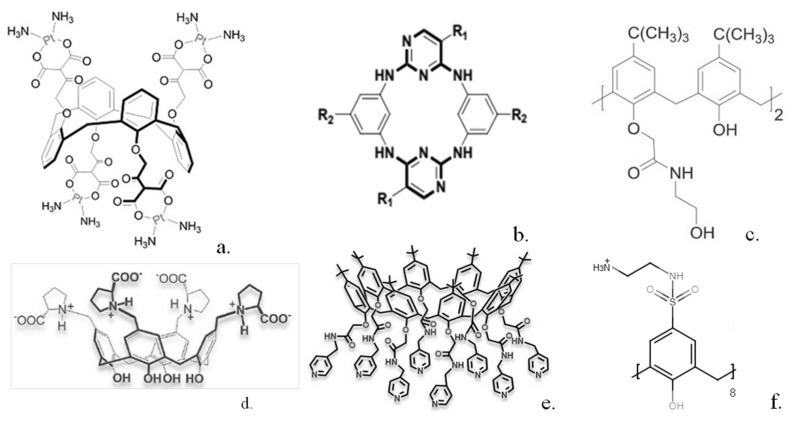
Structures of some calixarenes with anti-cancer properties (**a**) “calixplatin” (**b**) azacalix[2]arene[2]pyrimidines (**c**) hydroxyamine substituted calixarene (**d**) L-proline substituted calixarene (**e**) picolylamine substituted calixarene (**f**) sulphonamide substituted calixarene. Reproduced with permission from [26] (Copyright Taylor and Francis 2014) [28,30,31] (Copyright Elsevier 2016, 2020, 2020) and [32] (Copyright John Wiley and Sons 2020).

In other work patented in 2010, a range of calixarenes was studied and shown in some cases to display significant in vitro anti-cancer activity in cell lines corresponding to melanoma and especially human acute lymphoblastic leukemia cells with an IC_50_ of 7.33 μmol L^−1^ [24]. Calix(4)arenes modified with N-acetyl-D-glucosamine units were shown to be effective against mouse melanoma models when combined with keyhole limpet hemocyanin [25]. In other work, calix(8)arenes substituted with multiple ureido and N-acetyl-D-glucosamine moieties were synthesised and shown to inhibit C6 glioma cell proliferation and migration [26]. Acyclic and other analogues bearing fewer substituents were shown to be ineffective. Tumour growth was shown to be significantly inhibited over a 14-day time period. Platinum derivatives such as carboplatin have been extensively used in chemotherapy, so a calix(4)arene bearing four platinum moieties (Figure 2a) was synthesised [27]. Tests showed the calixplatin to have a superior anti-cancer activity to carboplatin when utilised against lung, breast and hepatocellular cell lines with an IC_50_ of 2.6 μmol L^−1^ against a non-small-cell lung cancer cell line.

A series of related compounds, azacalix[2]arene[2]pyrimidines (Figure 2b), were synthesised, and several of these restricted both proliferation and migration of human cancer cell lines, such as for example a pyrrolidine substituted derivative that showed an IC_50_ value of 0.58 μmol L^−1^ against the MCF7 cell line [28]. A number of calix(4)arene derivatives containing acyclic and macrocyclic amide substituents were also synthesised and demonstrated antiproliferation activity against MCF-7, LNCaP and U87 cell lines at low μmol L^−1^ concentrations [29]. A range of other substituted calix(n)arenes have also been used as anti-cancer agents. Calix(4, 6, 8)arenes substituted with hydroxyamine units were used against a range of cancer cell lines such as human ovarian cancer cells [30]. For some of these, such as that shown in Figure 2c, IC50 values ranging from 1.6 μmol L^−1^ to 11.3 μmol L^−1^ were obtained. More recently, calix(4)arenes bearing L-proline groups (Figure 2d) and calix(8)arenes bearing picolylamine groups (Figure 2e) were synthesised by the same group. Some of these calix(4)arenes [31] were utilised against the DLD-1 colon cancer cell line and displayed IC_50_ values ranging between 29.35 and 64.57 μmol L^−1^. The calix(8)arenes substituted with picolylamine groups [32] also displayed cytoxicity against the same cell line with IC_50_ values of 3.96–6.12 μmol L^−1^. A series of calix(4)arenes bearing sulphonamide groups (Figure 2f) have been synthesised [33] and demonstrated growth inhibition against human cancer cell lines MCF-7 and MIA PaCa-2.

**Figure 3:**
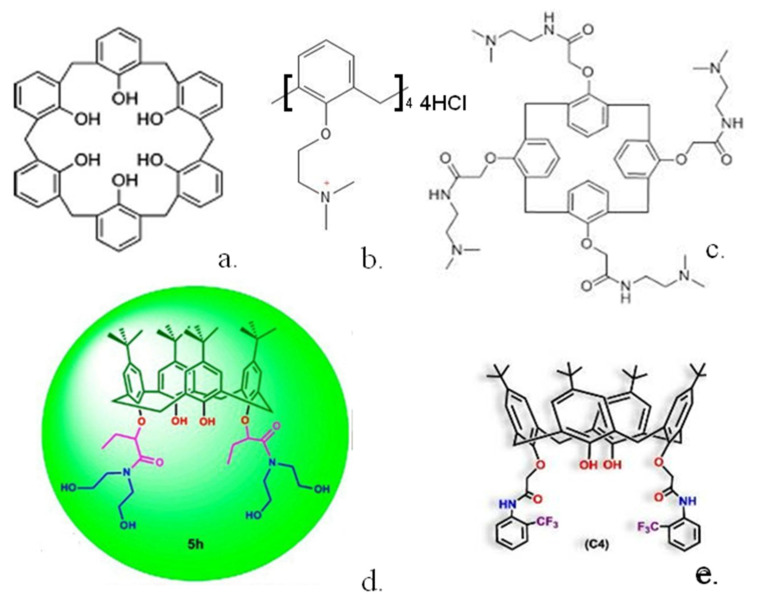
Structures of some calixarenes with anti-cancer properties (**a**) calix(6)arene (**b**) ammonium substituted calix(4)arene (**c**) calix(4)arene 0118 (**d**) calix(4)arene diamide (**e**) trifluoromethyl aniline substituted calix(4)arene. Reproduced with permission from [39] (Copyright American Chemical Society 2012) and [41,42] (Copyright Elsevier 2021).

In other interesting work, a simple unsubstituted calix(6)arene [34] (Figure 3a) was shown to greatly reduce human pancreatic cancer cell viability, whereas much smaller effects were shown for substituted calix(6)arenes or similar calix(4 and 8)arenes. The calix(6)arene was shown to abolish signal transduction of tyrosine kinase receptors within the cells. Further work [35] indicated that the calix(6)arene specifically interacts with the enzyme binding site and may well be effective in treating patients with high expression of the AXL protein.

One requirement for many tumours to grow is the formation of new blood vessels to supply oxygen and other nutrients to the tumour. A range of calixarenes have been utilised, which inhibit this procedure. For example, platelet-derived growth factor has been shown to be implicated in angiogenesis, and a series of modified calix(4)arenes were synthesised, which bind to the three-loop region of this species with a range of affinities, inhibiting its binding to its receptor [36]. The three most potent inhibitors gave IC_50_ values from 0.11 to 0.43 μmol L^−1^ in mouse NIH 3T3 cells. Angiogenesis can be inhibited by a series of calixarenes [37] as demonstrated by tests utilising human ovarian cancer or mouse melanoma cell lines. Further modification of one of the most potent calixarenes gave a simple calix (Figure 3b), which was much more effective at inhibiting cell growth in several human cancer lines [38]. Another compound, calix(4)arene 0118 (Figure 3c), a mimetic of the angiostatic amphipathic peptide Anginex [39] was also shown to be highly effective. This calix(4)arene was shown to bind to the galectin-1 protein and inhibit tumour growth in mice.

Two other similar calix(4)arenes, in which click chemistry had been used to radio-label the macrocycle with ^18^F, were synthesised [40] to allow radio tracing of their uptake. Non-radioactive analogues were shown to be as effective or up to 10 times more effective than the basic calix(4)arene in human ovarian carcinoma cells. A range of calix(4)arenes substituted with amide groups have also been synthesised [41] and utilised against human lung, breast, colon and hepatocellular carcinoma cells. A disubstituted calix(4)arene (Figure 3d) was shown to display the highest inhibitory effects whilst displaying minimal inhibition towards healthy cells. More recently, calix(4)arenes substituted at the lower rim with trifluoromethyl aniline groups [42] were tested against healthy and cancer cell lines. The calixs were shown to have strong anti-proliferative activity; for example, one compound (Figure 3e) was 25 times more effective against MCF-7 breast cancer cells than against healthy Vero kidney cells. Calix(4)arenes could also be modified with pyridinium units [43] and shown to display preferential cytoxicity against MCF-7 cell lines over a range of 25 to 100 μg/mL.

Li–Fraumeni syndrome is a hereditary disorder that leads to the formation of multiple tumours and is thought to be due to a mutation of the TP53 gene, which encodes the p53 tumour suppressor protein. Calix(6)arenes bearing either six pyrazole or six imidazole units have been shown to stabilise the structure of the protein and improve its transcription, pointing to its potential use in cancer therapy [44]. Another possible weapon against cancer is to utilise the patient’s own immune system. Tn moieties [45] are glycosyl units that are overexpressed on the surface of tumour cells. Calix(4)arenes could be synthesised with each calix molecule bearing four of these species. Immunisation of mice with this material leads to a high production of anti-Tn antibodies, which could potentially attack cancer cells. Further work by the same group substituted calix(4 or 8)arenes with PDTRP units, a peptide that is found in the MUC1 protein, which is overexpressed in human epithelial carcinoma [46]. The calix(8)arene was shown to provoke a much higher immune response in mice than the corresponding calix(4)arene.

One advantage of using calixarenes is their ability to undergo chemical or other transformations depending on the conditions they encounter. This property allows the construction of calixarenes that respond to their environment, thereby making their behaviour selective. Hypoxic conditions are often found within tumours and this led workers to synthesise an azo-substituted calix(4)arene [47], which degrades under hypoxic conditions in vivo. This calixarene demonstrated strong host–guest interactions with a range of imaging dyes and anti-cancer drugs and, in mouse models, transported its cargo selectively into the tumours. In other work [48], multiple azo-calix(4)arenes could be grafted onto bovine serum albumin moieties. If a mixture of calixarene-drug host–guest compounds were utilised, this would allow precise loading of these components onto the protein. This could then be applied to mouse models, allowing simultaneous delivery of two or more drugs into the tumours. Since this calixarene binds strongly under normal conditions to a wide range of targets and releases them under hypoxia, this allows for the development of combination chemotherapy and potential personalised treatment. Other in vivo studies demonstrated that a simple injection of the calix(4)arene host–guest compounds allowed them to bind with in situ albumin [49]. The resultant ternary complex demonstrated increased blood circulation times and higher accumulation of the drug within the tumours compared to the free drug, sulphonated cyclodextrin carriers and other formulations.

Pillarenes are a much more recent addition to this family of macrocyclic compounds. They have been shown to have a number of potential medical applications, especially in the field of theranostics as reviewed here [50]. At present, there have been few reports on the use of pillarenes in cancer detection and treatment, although they have been shown to demonstrate high selectivity towards binding certain amino acid units such as lysine [50]. In one study, both an alkoxy-substituted (5)pillarene as well as a sulphonated calix(4)arene were tested for their activity against human papilloma virus, one of the leading causes of cervical cancer [51]. Both species were shown to bind to the virus, with the pillarene being shown to display higher inhibition activity than the calixarene.

Recent work has investigated the use of environmentally responsible pillarenes as selective drug release agents that target cancer cells. For example, a carboxy-substituted pillarene [52] could be synthesised that would form a stable complex with oxiplatin. The complex had a much lower dissociation constant in acidic media (such as often found in tumours) than at physiological pH. The complex was found to inhibit tumour growth in mouse models. Similarly, it proved possible to modify pillarene with galactose units and constructed nanoparticles of this material complexed with camptothecin [53]. On application, these nanoparticles were found to be stable under physiological conditions. However, when tested under simulated tumour conditions, the high levels of glutathione found under those conditions led to competition with and displacement of the drug from the complex. The galactose units also led to preferential incorporation of the complex in HepG2 cells. A pyridinium-substituted pillarene could be combined with sodium dodecyl sulphate in water to make vesicles capable of incorporating doxorubicin [54], and these vesicles preferentially accumulate in tumour tissue. The high levels of adenosine triphosphate inside tumour cells led to the competitive displacement of the dodecyl sulphate from the pillarene, disintegration of the vesicles and release of the drug. Both in vitro and in vivo tests showed improved drug efficiency and a reduction in systemic toxicity.

## 3. Cyclodextrins

**Figure 4:**
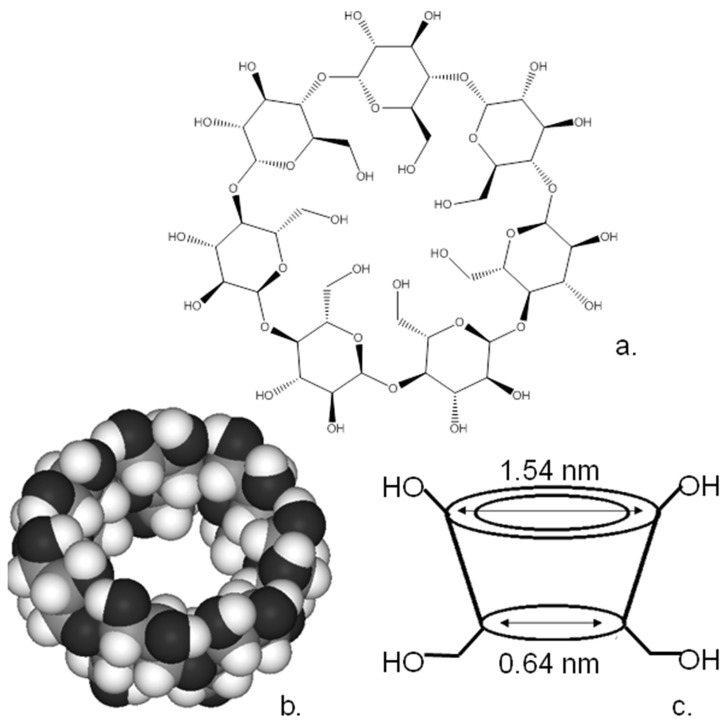
(**a**) Structure, (**b**) crystal structure and (**c**) schematic of cyclodextrin.

Cyclodextrins are a family of macrocyclic polysugars [5]. Although a wide range of ring sizes have been synthesised, the most common variants of these moieties are α, β and χ-cyclodextrins, which contain 6, 7 and 8 α-1,4-d-glucopyranoside units, respectively, in the macrocyclic ring. Figure 4 shows the chemical formula and X-ray crystal structures of β-cyclodextrin. As can be seen from the schematic of the cyclodextrin structure (Figure 4c), the cyclodextrin forms a bowl-shaped structure with a central cavity, resembling the calixarenes described earlier. These materials have a hydrophilic external surface and relatively hydrophobic interior hydrophobic cavities from 0.5 to 1.0 nm in diameter, meaning that they are highly efficient receptors, especially for hydrophobic guests in an aqueous environment [5].

The presence of reactive hydroxy groups on the cyclodextrin rims makes them highly suitable for chemical modification, and a wide range of cyclodextrin derivatives have been synthesised. What is also important is that the reactivity of the hydroxyl groups within the cyclodextrin varies, allowing for specific modification of these groups and, therefore, the controlled substitution of the cyclodextrin macrocycle as described in detail here [5]. An initially controlled substitution of the macrocycle would appear to be an insurmountable problem. Fortunately, the hydroxyls of the macrocycle are not absolutely identical. The hydroxyls at the 3-positions of the sugar moieties are more difficult to access than their counterparts. Also, the most basic and nucleophilic of the hydroxyl groups are those located at the 6-positions of the sugar moieties whilst those at the 2-position are the most acidic hydroxyls. This variation in the properties of the hydroxyl groups has allowed the development of a range of reaction schemes, allowing for the controlled substitution of some or all of the hydroxyls of the cyclodextrins and the substitution of different types of hydroxyl with differing functional groups as reviewed here [5]. This selective functionalisation along with the ease of varying the ring size means that these moieties can be used for a wide range of specific applications [5]. The ability to control their molecular structure has enabled their utilisation as sensing agents and drug carriers in a range of anti-cancer applications that have been extensively reviewed elsewhere [55,56,57,58]. Although there is a range of cyclodextrin sizes, the commonest form and the one most used in the applications below is β-cyclodextrin, since this is the easiest and least expensive to produce [55]. 

### 3.1. Sensing Applications

As with the calixarenes, it has been shown possible to utilise cyclodextrins for detecting cancer cells and biomarkers. Multiwall carbon nanotubes [59] could be used as a substrate for grafting β-cyclodextrin and, combined with an electrochemical probe, tetrathiafulvalene (TTF) carboxylate to construct electrodes capable of detecting cancer cells such as liver cancer cells SMMC-7721 and HepG2, leukemia K562/B.W K562/ADM cells, with a detection limit of 10^3^ cells mL^−1^.

In other work, β-cyclodextrin units could be immobilised onto electrode surfaces. The interaction between the macrocycle and ferrocene guests was shown to be controllable by electrochemically oxidising and reducing the ferrocene moiety [60]. This interaction combined with the use of a folic acid linker to bind to cancer cells enable the selective capture and release of these cells with a minimum detection limit of 10 cells. A similar cyclodextrin-ferrocene interaction was used in conjunction with ferrocene-labelled gold nanoparticles modified with aptamers for the breast cancer marker HER2 [61], thereby allowing the electrochemical detection of the target with a detection limit of 4.9 ng mL^−1^.

A number of chemically modified cyclodextrins where the hydroxyl groups were selectively substituted with either amino, benzyl or mannose groups could be immobilised via non-covalent interactions to form a composite with the 2D form of carbon, i.e., graphene [62]. These composites could be sprayed onto interdigitated electrodes and served as electrochemical detector arrays for the determination of a range of organic compounds that are often found in the breath of cancer sufferers. The resultant “electronic nose” was capable of detecting cancer marker volatile organic compounds such as benzene at levels as low as 400 ppb. Nanocomposites could also be made of CuO_2_/graphene oxide/β-cyclodextrin or ferrocene carboxylic acid/graphene oxide/β-cyclodextrin and used as substrates to immobilise antibodies to the cancer biomarkers alpha-fetoprotein (AFP) and carcinoembryonic antigen (CEA) [63]. Graphene oxide/gold nanoparticle-modified electrodes could then be used to develop a sandwich-type electrochemical immunoassay for these cancer marker proteins. The resultant assay was capable of detecting these moieties with linear ranges from 0.001 to 80 ng mL^−1^ for AFP and CEA with detection limits of 0.2 pg mL^−1^ and 0.1 pg mL^−1^ for AFP and CEA, respectively.

Cyclodextrins have also been utilised within the field of cancer imaging. The potential for combining the imaging properties of various nanoparticles with the sensing ability of cyclodextrins has been recently reviewed [57]. For example, spherical gadolinium oxide nanoparticles (75–95 nm in size) could be coated with a β-cyclodextrin copolymer and folic acid units [64]. The resultant composites displayed high blood compatibility and minimal cytotoxicity towards normal human breast cells and allowed magnetic resonance imaging (MRI) studies to be performed. This method allowed in vitro imaging of cancer cells and acted as an in vivo MRI contrast agent for imaging of tumours in mice. A similar method was used where superparamagnetic iron oxide nanoparticles coated with crosslinked curcumin were synthesised. When injected into mice these materials acted as excellent contrast imaging agents, allowing resolution of tumours 4–8 h after injection [65]. The use of cyclodextrins as scaffolds for the substitution and assembly of MRI contrast agents has been recently reviewed [66] and the modification of these agents by either covalent linkages or by forming inclusion compounds with cyclodextrins has been discussed.

Cyclodextrins can be used as imaging agents by modifying them with suitable dyes. This tends to be by forming inclusion complexes of dyes encapsulated in cyclodextrins, some of which will be discussed later since this procedure tends to be simpler than covalent modification. However, cyclodextrin/dye compounds can be synthesised, such as a β–cyclodextrin/cyanine dye compound [67] where “click” chemistry could be used to graft a single dye unit onto a β–cyclodextrin unit, the resultant compound being able to be imaged by confocal laser scanning microscopy in HeLa cells and also shown to act as a host for doxorubicin.

A highly fluorescent composite of β-cyclodextrin with gold nanoclusters could be synthesised and was shown to be highly biocompatible. The composite was selectively taken up by gastric cancer cells even in the presence of normal gastric cells [68]. The strong red fluorescence of the composite combined with dark-field imaging demonstrated that they were not only selectively taken up by the cancer cells but also had penetrated into the cells and were not just bound on the surface. Other workers utilised poly(p-phenylene-β-cyclodextrin)-graft-poly(ethylene glycol) modified gold nanoparticles to image U87 glioblastoma cells [69]. The resultant fluorescent complex was shown to image U87 cells with high selectivity over Vero cells. The poly(ethylene glycol) sidechains on the composite conferred high solubility and biocompatibility onto the composites, leading to minimal cytostatic and cytotoxic effects. Their high solubility allowed extensive uptake by the U87 cells, leading to high-quality imaging. Organic dyes could also be utilised; spherical nanogels of cyclodextrin/spiropyran/4-amino-7-nitro-1,2,3-benzoxadiazole composite (200 nm diameter) were formulated [70]. Depending on the illumination, these could fluoresce red or green. Selective imaging of Cal27 human tongue cancer cells was possible using these materials.

One major issue with cancers is that cells can break off tumours, so-called circulating tumour cells, and then be transported by the bloodstream to other parts of the body, giving rise to metastasis. Electroluminescent sensing could be utilised to detect these biomarkers [71], whereby a glassy carbon electrode could be modified with a gold nanoparticle/cyclodextrin/ruthenium complex/graphene composite. Ferrocene-labelled aptamers could be then immobilised on the surface of this composite. The ferrocene units interacted with the ruthenium complex, quenching its electroluminescence. When exposed to tumour cells, binding occurs between the cells and the aptamers, and the resulting change in aptamer conformation causes the ferrocene to move away from the electrode, preventing its quenching effect and allowing electroluminescence to occur. This method allowed highly selective detection of the cancer cells with a detection limit of 40 cells per mL^−1^.

### 3.2. Medical Applications

Cyclodextrins have been shown to have minimal oral toxicity since they are not adsorbed but instead simply “pass through” the gastrointestinal system and are excreted [72]. A number of cyclodextrin compounds have also been shown to be safe when administered by injection, although there are some that display adverse nephrotoxicity [73]. Reports have been produced that give suggested thresholds for the safe administration of cyclodextrins by various routes [74].

Their low toxicity has led to interest in the possibility of using cyclodextrins and substituted analogues as anti-cancer agents. The widest application is for use as transport agents for a variety of anti-cancer drugs due to their abilities to both solubilise these often poorly water-soluble drugs and stabilise them in conditions such as in the gastrointestinal tract. Many of these host–guest complexes of cyclodextrins with a wide range of drugs have been studied; however, their inclusion is outside of the range of this review, but a number of other extensive reviews on these systems have been published [55,56,57,58].

Substituted cyclodextrins have been shown to have marked anti-cancer properties. Methylated cyclodextrins can display higher water solubility than their parent compounds [5] and interact strongly with cell membranes. For example, methylated cyclodextrin was shown to bind to and extract cholesterol from cell membranes [75]. A number of human cancer cell lines were treated with cyclodextrin, which led to decreases in transmembrane potential and DNA content in these cells. When applied by intratumoural injection to mice, there was a dramatic inhibition of tumour growth, indicating potential applications of this material as an anti-cancer agent. In other work [76], a number of substituted cyclodextrins were used and methylated derivatives were shown to induce apoptosis of cancer cells—again by extraction of cholesterol from their cell membranes. The cytotoxicity of the methylated compounds was shown to be higher than that exhibited by the parent cyclodextrins or their sulphonated derivatives. Methylated cyclodextrin was also shown to display good anti-cancer activity when utilised against MCF7 breast carcinoma and A2780 ovarian carcinoma cell lines [77]. When applied by injection to mice grafted with human tumours, the cyclodextrins were shown to accumulate within the tumour and reduce tumour growth by at least a factor of two compared to a control group. 

**Figure 5:**
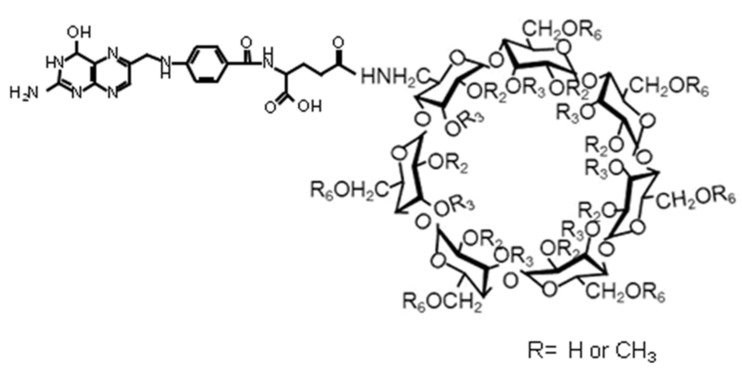
A folate-substituted cyclodextrin displaying anticancer activity. Reproduced from [79] with permission from Springer (Copyright Springer 2013).

In an attempt to improve the specificity of the cyclodextrins, a methylated cyclodextrin was modified by substitution with a folic acid unit (Figure 5) in an attempt to target the folate receptors often overexpressed by cancer cells [78]. The resultant compounds displayed potent cytotoxicity when compared to the parent methylated cyclodextrin in KB cells, although not in A549 cells. In further work, the same compound was applied by intratumoural or intravenous injection to mice bearing FR-positive Colon-26 cells [79]. The anti-tumour activity of the folate-derivative at a level of 30 mg kg^−1^ was shown to be higher than the same dose of methylated cyclodextrin or the common anti-cancer drug doxorubicin. All of the mice treated with methylated cyclodextrin or doxorubicin died within 70 days, whilst all the mice treated with the folate-derivative survived for at least 140 days. 

In other work, the simpler methylated cyclodextrin was shown to remove cholesterol from plasma membranes, and, in conjunction with benzyl isothiocyanate, it demonstrated enhanced cytotoxicity against human colorectal cancer cells [80]. Similar effects could be obtained using methylated cyclodextrin along with the anticancer drug tamoxifen; the cyclodextrin appeared to enhance the uptake of tamoxifen into melanoma cells and increased the cytotoxic effects of this drug [81].

There have also been a number of more recent papers published on the use of folate-appended β-cyclodextrins against a number of other cancers. Lhara cells, which are human melanoma cell lines that express folate receptors, could be treated with a folated methylated cyclodextrin [82]. The folated cyclodextrin entered the lhara cells and displayed cytotoxic effects as well as caused the formation of autophagosomes within the lhara cells, whereas a simple methylated cyclodextrin did not. Melanoma growth was suppressed in mouse models, suggesting the potential of this compound as a chemotherapy agent. In more recent work [83], folated-cyclodextrin was shown to be cytotoxic towards epithelial ovarian cancer cells, especially when combined with paxitaxel, and to reduce tumour growth in mouse models. The same cyclodextrin has been shown to be effective against folate receptor-carrying ES-2 (RFC+) ovarian cancer cells in mouse models [84], with cyclodextrin displaying high cytotoxic effects against the cancer cells.

Another cyclodextrin that has been used in medical applications is hydroxypropyl β-cyclodextrin, which has been approved for oral, buccal, rectal, ophthalmic and intravenous application [55]. This compound displays very low toxicity [85], with mice being able to tolerate intraperitoneal dosages of up to 10,000 mg kg^−1^. Again, this compound displays a high affinity for cholesterol, which has led to its investigation as a possible anti-cancer agent [86]. Hydroxypropyl β-cyclodextrin was shown to inhibit the proliferation of a number of leukemia cell lines by removing cholesterol from the cell membranes leading to apoptosis and was also effective against cell lines containing the T315I BCR-ABL mutation, which makes them resistant to tyrosine kinase inhibitor drugs. Intraperitoneal injection of hydroxypropyl β-cyclodextrin to leukemia mouse models significantly improved their survival, whilst application to healthy mice controls showed no significant adverse effects. What makes this compound especially important is that it acts against a range of leukemias including acute myeloid leukemia, acute lymphoblastic leukemia and chronic myeloid leukemia as well as inhibiting hypoxia-adapted cells. The compound has also been approved for treating a lysosomal lipid storage disorder, Niemann-Pick Type C disease [87].

In recently published work, hydroxypropyl cyclodextrin has been shown again, probably by its cholesterol binding and depletion ability, to both impede breast cancer cell growth and cause cancer cell death [88]. Experiments using mouse xenografts have proved extremely promising, showing total eradication of early-stage tumours and what the authors describe as remarkable reductions in intermediate and late-stage tumours.

Substitution of hydroxypropyl cyclodextrin with a folate group as described for the materials above could be performed to provide a material with the capability to cause autophagic cell death in chronic myeloid leukemia cells, especially when combined with imatinib, an ABL tyrosine kinase inhibitor [89]. In mouse models, this combined therapy had a much stronger inhibitory effect on cancer progression than either component separately. 

Folate-substituted cyclodextrins have also been very effectively used as drug carriers; again, this is outside of the scope of this review, but there are many examples in the literature, some of which are summarised here [90].

Cyclodextrins have also been used to construct “responsive” materials, which can undergo physical or chemical transformations in response to a stimulus or the nature of their environment. These materials will be discussed elsewhere in the review, but one example is where β-cyclodextrin was reacted with a difunctional crosslinking agent containing a disulphide linkage to give a crosslinked nanocage capable of hosting doxorubicin [91]. This composite showed good stability and biocompatibility in vivo and was selectively adsorbed into tumour cells where the high levels of glutathione in the tumour led to a reduction in the disulphide bond to thiols. This caused the nanocage to disintegrate, releasing the drug, and mouse models showed enhanced tumour suppression and improved survival rates compared to those treated with doxorubicin.

The results of the research above demonstrate the possibility of using substituted cyclodextrins as anti-cancer drugs. However, toxicological issues may need to be addressed before use since, unlike the compounds described above, many substituted cyclodextrins often display higher toxicology than their unsubstituted parent compounds. 

## 4. Cucurbiturils

**Figure 6:**
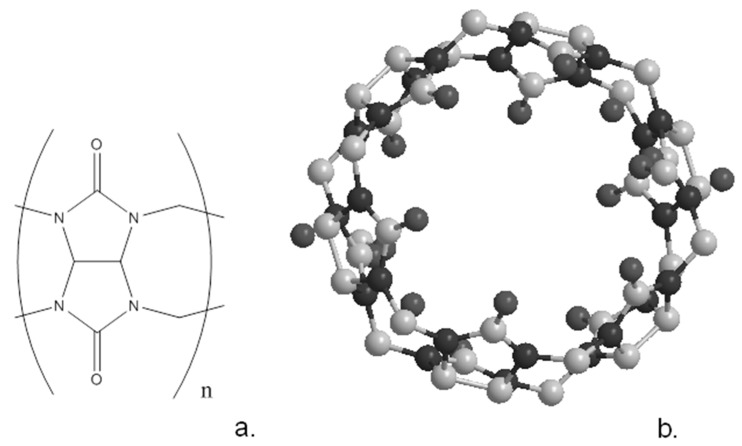
Schematic structure (**a**) of cucurbituril and (**b**) the crystal structure of cucurb(7)uril.

Cucurbiturils are a family of cyclic compounds obtained by the condensation of glycouril with formaldehyde [5]. As with the other macrocyclic compounds described earlier, they can be synthesised in a variety of ring sizes; the most common are the cyclic hexamer, heptamer and octamer, often known as CB6, CB7 and CB8 [5]. The schematic of the cucurbituril frame and the crystallographic structure of CB6 are shown in Figure 6. The shape of these molecules resembles a pumpkin, family *Cucurbita*, leading to their names. Table 1 lists the characteristics of the different cucurbiturils and shows how the central cavity increases dramatically in size with the incorporation of more glycouril units. Although these compounds are generally highly insoluble, they can be solubilised by complexation with metal salts while derivatives of the macrocycles can be synthesised with enhanced solubility.

As with the previously discussed arenes and cyclodextrins, the complexation ability of these macrocycles has led to them being utilised in a variety of fields, which include sensing [92,93], theranostics [93] and drug delivery [94], along with a number of other potential medical and biochemical applications [94,95]. A comprehensive discussion of the many potential uses of cucurbiturils CBn (where n is the number of glycouril units) is outside of the scope of this article, but a number of recent reviews have been published [92,93,94,95]. Cucurbiturils have been shown to display strong complexation abilities, while also displaying low cytotoxicity and an ability to penetrate cell membranes [94].

Cucurbiturils have been shown to be of application in the fields of recognition and imaging. For example, a CB7 could be substituted with a rhodamine dye with the conjugate being shown by confocal fluorescence microscopy to be adsorbed into HT22 neuron cells [96]. In other work, CB8 substituted with a biotin unit could be used to complex toluidine blue dye [97]. Quenching of the dye by CB7 removed its fluorescence; this conjugate could be selectively taken up by cancer cells, whereupon the dyes were released from the conjugate, regaining their fluorescence and thereby allowing for cell imaging. When mice were treated with this complex and then irradiated, the dye displayed its known phototoxicity, killing most of the tumour cells, whereas only a minimal amount of tumour apoptosis/necrosis was observed in mice treated with just the dye.

A ferrocene-CB7 complex could be used as an electrochemical marker within a sandwich immunoassay and DNA barcoding protocol for a range of cancer biomarkers, allowing for the detection of marker proteins such as alpha-fetoprotein at potentially subfemtomolecular levels [98]. Combining this technique with a variety of sandwich immunoassays allowed the determination of a number of biomarker proteins including prostate-specific antigen, neuron-specific enolase, alpha-fetoprotein, carcinoembryonic antigen and carbohydrate antigen 19-9 [99].

Other workers developed a sensor capable of detecting the DNA associated with a breast cancer susceptibility gene. This system used a dual DNA sensing system, utilising a target single-stranded DNA that could simultaneously hybridise with ferrocene-labelled DNA/Au nanospheres (FcNS) and horseradish peroxidase-labelled DNA/Au nanospheres. The resultant complex was adsorbed at an electrode modified with CB7 and allowed electrochemical determination of the target with high specificity and a detection limit of 25 pmol L^−1^ [100]. Other workers developed a method for the determination of microRNA-182-5p, a prostate cancer biomarker [101]. Using DNA cross configuration-fuelled target cycling and strand displacement reactions, the target RNA could be amplified to give a large number of two tryptophan-labelled single-stranded DNAs [100]. These were then trapped on electrodes modified with CB8/methyl viologen complexes allowing electrochemical determination of the target. The resultant sensor displayed a linear range between the response and the log of micro-RNA concentration from 0.001 pmol L^−1^ to 500 pmol L^−1^ and a limit of detection estimated as 0.5 fmol L^−1^. Also, by resetting the potential, it proved possible to displace the DNA strands, allowing the sensor to be reused at least ten times without loss of performance.

In other work, cucurbituril could be used to improve the luminescence efficiency of gold nanoparticles from 7.5% from unsubstituted nanoparticles to 39% when modified by CB8 and 51% when modified by CB7 [102]. The CB units could bind to amino acid units immobilised on the nanoparticle surfaces and the red phosphorescence emitted by these composites allowed for the imaging of live A549 lung cancer cells. Gold nanoparticles could also be modified by a tetraalkyl ammonium substituted thiol to substitute them with cationic headgroups; these were then used to bind fluorescent protein units [103]. These nanoparticles quenched emission from red, green and blue fluorescent proteins. The addition of cell lysates released the fluorescent proteins allowing for fluorescence. However, when CB7 was added, lower levels of fluorescence were observed due to modification of this interaction. Thus, three output readings (red, green, blue) could be obtained with and without CB7, allowing for six outputs from a single sample well. Linear discrimination analysis allowed differentiation between normal, cancerous and metastatic cell lines. Potentially, this technique will allow the determination of cell tumourigenicity and invasiveness with small sample sizes of about 1000 cells.

Gold electrodes could be modified by thiol-substituted peptides, which specifically bind to the cancer marker CD44. These monolayers could be digested by trypsin; however, the binding of CD44 hindered this process [104]. After exposure to the test solution and trypsin, the CD44 could be removed by heat treatment; the surface peptides were then treated with CB8 to give a surface capable of binding gold nanoparticles modified with a diphenyl alanine unit. These nanoparticles acted as a catalyst for silver metal deposition, which could be determined electrochemically. The resultant sensor could determine CD44 with a detection limit of 2.17 pg mL^−1^ and CD44 positive breast cancer cells at a limit of 8 cells mL^−1^.

Gold nanostars could be modified with CB7 units to improve their stability and facilitate their ability to transport drugs. Irradiation with near-IR laser (808 nm, 2 Watts, 10 min) radiation could increase the temperature of a 12.5 pmol L^−1^ composite solution to 49 °C [105]. This combined approach in MCF-7 mouse models significantly prevented tumour growth. CB6 could also be combined with gold nanoparticles modified with a peptide specific for binding to the neuropilin-1 receptor found in some cancer cells [106]. This combination along with the anti-cancer drug curcumin formed self-assembled spherical nanocapsules, which specifically targeted neuropilin-1-rich cancer cells. CB5 was also shown to bind to tubulin. The resultant nanocapsules could cause microtubule depolymerisation and significant tumour regression in a mouse melanoma model. 

It has proved possible to combine more than one type of macrocyclic compound to give enhanced beneficial properties. For example [107], it proved possible to combine a CB7/adamantane diamine complex with a cyclodextrin-modified hyaluronic acid. This composite proved capable of transporting small interfering RNA specifically into malignant human prostate PC-3 cells. 

## 5. Other Recent Work

Within this review, we have concentrated on the simpler macrocyclic compounds such as calixarenes, cyclodextrins, etc.; however, there is a wide range of other complex macrocycles that are suitable for the treatment of a range of cancers. One recent review looked at 68 of the macrocyclic drugs available on the market and reported that 10 of them were being used to treat cancer [108]. Within this review, the synthesis and the various advantages and disadvantages of these drugs are reported. Other workers have recently reported on the use of a dynamic macrocycle where a thiol–disulphide reaction is used to generate macrocycles that are capable of transporting and delivering both doxorubicin and small interfering RNA [109]; whereas the macrocycles could achieve drug-loading contents of over 30%, linear polymer analogues proved incapable of drug binding. The macrocycle/drug/RNA composites proved effective against tumour cells in vitro.

## 6. Detection of Carcinogens

“Prevention is better than cure” can be applied to the field of cancer mitigation. Although cancer can be due to many causes, one of these can be exposure to carcinogenic compounds. In some cases, this exposure can be eliminated, for example by not smoking and minimising the potential for passive smoking. However, many carcinogens are present at low levels in the environment, and early detection and quantification can aid in reducing exposure and demonstrating the necessity for environmental clean-up.

There is a wide range of potential carcinogenic compounds, and we will provide just a brief overview of how macrocyclic compounds can aid in their detection. All the macrocycles mentioned earlier contain a central cavity and various substituents, meaning that thin films of these are usually highly porous in nature, allowing rapid diffusion of small molecules within the matrix and their reversible binding to these films. A wide variety of transduction methods can be used to analyse this binding, including electrochemical, optical and mass-sensitive methods.

Calixarenes have been widely used in the field of gas sensing and we will just give a few examples; for more detail, a series of reviews have been published [110,111,112]. As an example, formaldehyde is a carcinogen given off by many manufactured products, including cleaning products and furniture materials such as plywood and medium-density fibreboard, with the World Health Organisation recommending an exposure limit of 0.08 ppm over a 30 min period. A number of calixarenes and calixresorcinarenes could be coated onto mass-sensitive quartz crystal microbalance (QCM) transducers and used to determine formaldehyde levels with a pyrogallol-based tetramer being capable of measuring formaldehyde levels from 109–2721 ppm in air [113]. Although this level is not sensitive enough to monitor exposure limits, this sensor does show the potential for formaldehyde binding and could also detect the higher levels associated with leaks or spills.

A wide range of organic solvents are potentially dangerous to health and the environment, many displaying flammable, explosive, toxic, carcinogenic and deleterious environmental effects. There has been a great deal of effort expended into the construction of calixarene-based sensors for these materials [110,111,112]. For example, four different calixarene compounds were coated onto shear acoustic wave sensors and exposed to a range of 21 organic vapours including carcinogenic chlorinated and aromatic vapours with acetone giving the strongest result [114]. Other workers deposited a range of calixarene materials onto QCM crystals by drop-casting and Langmuir–Blodgett methods [115] and again exposed them to a wide range of vapours. Some films showed preferential sensitivity towards the carcinogenic solvent chloroform, whereas others showed a marked sensitivity towards aromatic solvents such as benzene and toluene, which are again carcinogenic. Aromatic solvents tended to be detected more effectively by the larger calix(6 or 8)arenes, and it was also found that mixtures of solvent vapours gave greater responses than the sum of their constituents, possibly indicating that different solvents bind in different locations in the calixarene matrix. Drop-cast films of a wide range of calix(4)arenes onto QCM crystals were also shown to display sensing properties towards a range of organic solvents, including chlorinated and aromatic compounds [116]. Dichloromethane was strongly bound by these systems, with one calix(4)arene demonstrating a detection limit of 54.1 ppm.

Self-assembly methods have also been used; for example, calix(4)resorcinarenes substituted with alkyl sulphide sidechains would spontaneously bind by multiple gold–sulphur bonds to gold surfaces [117]. These films showed a remarkably high affinity for the common dry-cleaning solvent perchloroethylene. Other workers deposited different calixarenes and resorcinarenes onto gold films and studied their interactions with several organic solvents. A wide range of compounds were used. The size of calixarenes has been shown to have an effect; LB films of calix(4, 6 and 8)arenes were deposited onto QCM crystals with the calix(8)arenes giving the fastest and largest response [118]. The same group also studied calix(4)arenes with between none and four *t*-butyl substituents; adding these groups gave the calixarenes larger surface areas at the air–water interface [119]. In all cases, chloroform gave the strongest response followed by benzene, toluene and ethanol, and of the calixarenes the tri-substituted compound gave the largest response. 

The same group at Baleiksir has also used surface plasmon resonance studies to study for example LB films of simple tetra-undecyl calix(4)resorcinarene on gold surfaces and their response to organic vapours, with chloroform again giving the strongest response [120]. They have also utilised a wide range of other calixarenes and calix(4)resorcinarenes, with much of this work having been reviewed recently [112]. Other workers also constructed composite materials of silica nanoparticles with polyallyl amine and infused them with calix(4 and 8)arenes bearing sulphonate groups [121]. These were shown to respond to paint vapours within five minutes of exposure, with the calix(4)arene giving the stronger response. A wide range of other calixarenes and related compounds have also been used to sense organic vapours as reviewed previously [110,112]. Calixarenes can also be combined with other measurement techniques to analyse environmental pollutants; a range of calixarenes, for example, could be mixed with polyurethane and polysulphone and spun to form nanofibres immobilised onto a stainless-steel wire [122]. This composite proved capable of extracting carcinogenic chlorobenzenes from water, which could then be determined by subsequent gas chromatography measurements with limits of detection as low as 0.1–1.0 pg mL^−1^ for a range of chlorobenzenes.

Cyclodextrins have also proven to be of use in the determination of a range of organic vapours and other carcinogens as reviewed here [123]. In early work, cyclodextrins modified with siloxane could be deposited onto QCM crystals and used to detect from 80 to 4 × 10^5^ mg m^−3^ benzene vapour in air with good selectivity over a number of other vapours [124]. Other workers synthesised β-cyclodextrins substituted with a fluorescent indolizine unit, and the resultant compound was shown to bind to benzene and toluene as well as phenolic compounds with a concurrent loss of fluorescence intensity [125]. The same method could also determine levels of pentachlorophenol as low as 10^−6^ mol L^−1^ [124].

Other workers have used fibre-optic sensors modified with cyclodextrins to detect the presence of carcinogenic polyaromatic hydrocarbon pyrene in contaminated water samples [126]. The pyrene moiety could be detected by its fluorescence, but this signal was increased by a factor of 14 by the cyclodextrin coating compared to an uncoated fibre with minimal interference from a range of common potential interferents. Fibres could also be coated with β-cyclodextrin immobilised in a polyvinyl chloride membrane and used to extract the carcinogen bisphenol A from water [127]. Fluorescence detection of the resultant complex allowed the determination of bisphenol A from 6.0 × 10^−6^–1.0 × 10^−3^ mol L^−1^ with a detection limit of 1.0 × 10^−6^ mol L^−1^. 

Competitive methods also proved of interest; fibres could be coated with polyvinyl chloride containing a cyclodextrin/pyrene complex, which is highly fluorescent [128]. However, the pyrene moiety could be displaced by bisphenol A, thereby quenching its fluorescence. This method allowed determination of bisphenol A from 7.9 × 10^−8^–1.66 × 10^−3^ mol L^−1^ with a detection limit of 7.0 × 10^−8^ mol L^−1^ and could be applied to the testing of landfill leachate. An interesting chemosensor was constructed where a cyclodextrin was substituted with a fluorescent naphthyl unit, which formed a self-inclusion complex where the naphthyl unit resided in the cyclodextrin cavity [129]. On binding guests, including chloroform and other chlorinated hydrocarbons, this complex was disrupted, forcing the naphthyl unit out of the cavity with resulting changes in its fluorescence.

Electrochemical methods have also been used. Both carbon paste and screen-printed carbon electrodes containing cyclodextrins with different sizes and substitution patterns could be constructed [130] and interrogated by differential pulse voltammetry. It proved possible to detect carcinogenic aminonaphthalene and aminobiphenyl compounds with typical ranges of 2.0 × 10^−8^–1.0 × 10^−6^ mol L^−1^ with detection limits of the order of 1.0 × 10^−9^ mol L^−1^. Later work [131] used carbon paste electrodes modified by cyclodextrins and DNA to determine levels of 1-aminopyrene and 1-hydroxypyrene from 2.0 × 10^−8^–4.0 × 10^−6^ mol L^−1^ with a detection limit of 1.0 × 10^−8^ mol L^−1^. The effects of the interaction between 1-aminopyrene and DNA could also be investigated.

It has proved possible to immobilise β-cyclodextrin and modified variants onto the surface of SPR chips and use them to determine levels of BPA and other phenols in water [132]. Also, other workers deposited nanocrystalline TiO_2_ onto QCM crystals [133], these porous films then being coated with β-cyclodextrin and used to detect o-xylene in the air. Irradiation of these systems with UV light produced oxygen radicals that destroyed any organic component and regenerated a fresh porous TiO_2_ film. More recently, cyclodextrins and their substituted analogues have been combined with graphene and used to construct electrochemical sensors capable of detecting a range of organic compounds, including volatile biomarkers for lung cancer [62]. Carbon nanotubes could also be utilised in similar work on developing electrical vapour sensors. The nanotubes were used as supports for polyamides containing a range of β-cyclodextrin derivatives [134], which could be deposited by a spray process. The resultant “electronic nose” was utilised to determine a range of volatiles, such as toluene, as well as to analyse volatile biomarkers for lung cancer.

The high insolubility of many cucurbiturils seems to have inhibited their application within the sensor field somewhat; however, these macrocycles, especially the smaller ones, appear to readily form complexes with many gases and organic compounds, indicating their potential use as gas or vapour sensors or sensors for water-born contaminants [135].

Nitrosamines are a series of potent carcinogenic compounds that exist in low concentrations in many foods and water supplies. Detection of the compounds at low levels could be obtained by exposing gold electrodes to a calix(4)resorcinarene tetrathiol that is bound to the surface by the formation of Au-S bonds to form a monolayer [136]. A model compound N-nitroso-N-butyl-N-propylamine could be adsorbed from water and then detected using adsorptive stripping voltammetry, allowing quantification of this species in solution down to concentrations of 10^−10^ mol L^−1^. In more recent work, polymers bearing calix(4)arene or *t*-butylcalix(4)arene tungsten–imido complexes could be immobilised onto QCM crystals and used to detect N-nitrosodimethylamine in air with limits of detection as low as 5 ppb [137]. 

Other workers synthesised a fluorescent cucurbituril derivative bearing naphthyl groups; this material was found to bind to a number of compounds including N-nitrosodimethylamine and other N-nitrosoamines found in tobacco smoke [138]. The fluorescent probe could be quenched by binding with metal ions; however, binding of the target N-nitrosoamines released this metal and restored fluorescence. By comparison of the cucurbituril with the behaviour of an acyclic control probe, it proved possible to quantify levels of nicotine (range 0–12 ppm; LOD: 0.75 ppm), N-nitrosonornicotine (range 0–18 ppm; LOD: 0.05 ppm), and 4-methylnitrosamino-1-(3-pyridyl)-1-butanone (range 0–21 ppm; LOD: 0.27 ppm).

Another potential use of macrocyclic compounds in cancer prevention is to use their selective binding abilities to remove carcinogenic species from the environment, thereby preventing the possibility of human exposure to these compounds. For example, calix(4)arene could be modified with a Mannich reaction at the upper rim and then copolymerised with p-dibromoxylene [139]. The resultant polymer was used as a solid-phase extraction for carcinogenic azo dyes such as Evans Blue or aromatic amines such as 1-naphthylamine from aqueous solutions. The affinity for the azo dyes was shown to be higher than that for the amine compounds. The polymer was shown to be more effective than its monomeric analogue. In other work, *t*-butylcalix(6 and 8)arenes were modified with ester or carboxylic acid groups [140] and then utilised to extract aromatic amines from aqueous solution. The *t*-butylcalix(8)arene substituted with carboxylic acid groups was shown to be the most effective extractant, probably because of its larger ring size and potential for electrostatic and hydrogen bonding interactions between acid and amine groups. 

The same group also substituted cyclodextrins with siloxane groups and then immobilised them by a sol–gel reaction onto magnetic iron oxide nanoparticles [141]. These could then be stirred with aqueous azo dyes, which could then be bound by the cyclodextrin units. Effective adsorption of the dyes was observed and easy removal of the nanoparticles from the solution could be attained by using an external magnetic field. Cyclodextrins have also been used to extract carcinogenic polyaromatic hydrocarbons from oil samples into aqueous solution [142]. Simply shaking a contaminated oil sample with an aqueous solution of χ-cyclodextrin led to the formation of cyclodextrin/aromatic complexes. Moderate to good extraction abilities were shown for different oil/contaminant mixtures. Some of the resultant complexes were highly fluorescent, enabling rapid and sensitive detection of aromatic contamination. Other workers also used a range of hydroxypropyl-β-cyclodextrin derivatives to extract crude oil from contaminated sands and soils to assess levels of alkane and aromatic hydrocarbon contamination and also to remediate these samples by extracting and removing the contamination [143].

## 7. Applications of Supermolecular Systems

Within this section, we will discuss what we think are the most promising applications for the materials and techniques described in the review. A wide number of macrocyclic compounds have been synthesised and their properties determined. Although the totally synthetic calixarenes, pillarenes and cucurbiturils all have their advantages, such as being able to be synthesised with very precise structures and substitution patterns, we feel that the most promising compounds currently are those based on cyclodextrins. Since they are based on natural products, the cyclodextrins tend to be biocompatible and display low toxicities. Their ability to be selectively modified and exist in a range of ring sizes also increases the range of their binding abilities. We will therefore discuss the potential uses of these materials.

### 7.1. Photodynamic Therapy

Photodynamic therapy is a technique where a suitable photoactivatable molecule known as photosensitiser can be applied to a patient. Upon irradiation with light of a suitable wavelength, this photosensitiser will adsorb the radiation and then undergo a reaction with naturally occurring molecular oxygen to generate reactive oxygen species. These species can be especially effective at destroying proteins, DNA and lipids in tumour cells. What makes this method potentially effective as a cancer treatment is if the photosensitiser can be selectively incorporated into cancer cells. This would allow for the treatment to be applied to the patient and after some time to allow the photosensitiser to migrate to and be adsorbed by the cancer cells; selective irradiation of the cancer site can be applied to generate reactive oxygen species, which will selectively destroy the cancer cells.

There are, however, several issues with this form of treatment. The photosensitiser dyes can be inherently toxic, non-selective, difficult to solubilise and unstable. Cyclodextrins have been studied as potential hosts for these dyes [144,145] since they could improve upon these characteristics. Many of the photosensitisers used are based on porphyrin-type molecules [144], for example, the first clinically approved photosensitiser Photofrin. A range of other photosensitisers have also been clinically approved for the treatment of cancer [144]. Cyclodextrins have been widely studied as potential hosts for photosensitisers and much of this work has been reviewed [145,146]. Cyclodextrins have already been shown to improve the efficiency and selectivity of photosensitisers by increasing their solubilities and reducing toxicity. 

Although cyclodextrins appear to be the most promising candidates for this therapy, other macrocycles have been used, for example, in this work where a photosensitiser dye was combined with a water-soluble calixarene [147]. This complex accumulates in the mitochondria of tumour cells. The toxicity of the photosensitiser was suppressed by complex formation. After accumulation, a second compound, benzidine dihydrochloride, was injected, competing with and releasing the photosensitiser that could then be activated by irradiation, and successfully kill cancer cells in vitro and in vivo.

Combined phototherapy/chemotherapy approaches also show high promise for the treatment of cancer. For example, cyclodextrins, in which the hydroxy groups had been reacted to give boronate esters, could be further substituted with polyethylene glycol chains and then used to form micelles, which were loaded with doxorubicin and the photosensitiser purpurin [148]. Upon intravenous injection into mice, these micelles were preferentially taken up by tumour cells. When irradiated, the photosensitiser generated high levels of reactive oxygen species, which cleaved the boronate linkages, causing the micelles to break up and release their cargo. In mice, this treatment led to the suppression of tumour growth with no observable toxicity effects. In other work [149], a porphyrin photosensitiser could be substituted with four cyclodextrin units by way of click chemistry. This material could be assembled into a framework using a linker unit containing two adamantane units that bind to the cyclodextrins and a thioketal linkage. Using this network improved the porphyrin solubility and reduced the quenching effect. The network could be loaded with doxorubicin and in vivo was found to be adsorbed by tumour cells. Confocal microscopy of cells showed the networks bound to the mitochondria. Irradiation led to the generation of reactive oxygen species that cleaved the thioketal link, destroying the network and releasing both drug molecules. Again, tumour suppression in mice was demonstrated. 

We feel the next steps will be to use the cyclodextrins to improve selectivity, i.e., by preferentially locating the CD/photosensitiser at the surface of or within the target cancer cells to reduce the amount of photosensitiser needed and minimise damage to healthy cells. Such approaches could for example utilise the folate-substituted cyclodextrins described earlier in this work.

### 7.2. Imaging

Another field in which cyclodextrins are being intensively studied is that of imaging of cancers [150]. Substituted cyclodextrins such as the methylated β-cyclodextrin and hydroxypropyl-β-cyclodextrin mentioned earlier in this review have been widely applied in this field. Although the cyclodextrins themselves are unsuitable for imaging, they can easily be substituted with a range of suitable labels, combining the desirable qualities of cyclodextrin and the activity of the label. 

The commonest labels are fluorescent species such as dyes, which can either be incorporated into the cavity or attached by covalent bonds [150,151]. Another method is radiolabelling where cyclodextrins containing ^14^C atoms could be synthesised or alternatively the positron emitter ^68^Ga could be complexed with cyclodextrins. This could be applied to the patient and its distribution analysed by positron emission tomography. Magnetic resonance imaging is also a possible technique and cyclodextrin-based nanoparticles have been investigated as MRI contrast agents as reviewed here [66]. A recent review lists and describes a range of labelling methods for cyclodextrins and their use in cancer imaging and treatment [150].

Since cyclodextrins can be synthesised to specifically bind to certain species, this allows the imaging and determination of the presence of these within or at the surface of developing tumours. This in vivo imaging will facilitate greater knowledge about neoplastic transformations of the tumour as well as its proliferation rate. The genetic or immunohistochemical profile of the primary tumour and its metastases could also potentially be determined. This will allow a much more tailored specific treatment for each case to be applied, potentially greatly improving its efficacy.

### 7.3. Other Applications

Theranostics can be defined as a technique where one drug (often radiolabelled) is used to identify a tumour and a second drug to destroy it. Cyclodextrin-based nanoparticles have been investigated as potential hosts for these drugs as reviewed recently [152]. A wide range of nanoparticles containing cyclodextrins have been utilised against a range of cancer cells including breast, lung, cervical and colon cancers [152]. The use of cyclodextrins has been shown to allow drug-loaded nanoparticles to selectively penetrate cancer cells and cause damage and trigger cytotoxic mechanisms leading to cell death.

Another recent review [153] also details many of the potential applications of cyclodextrins as components in building nanocomplexes suitable for imaging, drug delivery and immunotherapy. Again, the advantages of cyclodextrins are detailed, such as targeted delivery and slow, sustained release of genetic material or other drugs. Other applications such as organelle imaging, controllable release of therapeutic contents, ease of functionalisation and the potential to incorporate them in other nanostructures are all detailed.

## 8. Conclusions

Within this review, we have endeavoured to provide an overview of recent work on using the most common macrocyclic families of compounds for the prevention, diagnosis and treatment of cancer. They display excellent sensing abilities for a wide range of guests such as carcinogens and cancer biomarkers and can be selectively tuned for certain guests by facile chemical modification. Some of these also specifically bind to cancer cells and can display drug-like properties. Another major field of use for these compounds is as transport systems for a wide variety of drugs; we have not covered this extensive field but have recommended several excellent reviews [15,18,19,20,46,48,49,50,51,154]. Each family of compounds has its own advantages and disadvantages. 

One major issue with the calixarene and cucurbituril families is their insolubility in water. However, this issue can be addressed by substituting the parent macrocycles with hydrophilic groups. Cyclodextrins, fortunately, are inherently much more amenable to dissolution in biological systems. Toxicology may also be an issue, and there must be an extensive study of this field before any use of these compounds in vivo. Stability may also be an issue, and the macrocyclic compounds must be stable in biological systems long enough to fulfil their purpose and also until they are excreted. Alternatively, if they do break down in the body, the resultant products must be harmless. The stability of these systems is less important in sensing applications since, in most cases, testing is undertaken on samples such as blood or urine, which have already been removed from the patient. 

Selectivity may also be an issue and must be considered, although many of the compounds discussed are inherently more cytotoxic to cancer cells than to healthy cell controls. Of special interest is the potential for easy functionalisation of the macrocyclic compounds with units such as folates, enabling selective targeting of cancer cells. Due to the multifunctional nature of the macrocyclic compounds discussed, it should prove possible to attach several of these units to the macrocyclic skeleton, thereby potentially increasing selectivity.

Calixarenes are highly promising because of the facile modification and inherent multifunctionality of these species. Although currently there are no clinical examples of approved calixarene-based prodrugs to date, there is the potential for widespread application in the future. Cyclodextrins display excellent biocompatibility and low toxicity, which makes them attractive targets for cancer applications. There are a number of cyclodextrin-based drug formulations currently on the market and this number is constantly increasing, although as yet none of these are for cancer [58]. Cucurbiturils have also shown some promise although solubility issues amongst others need to be addressed before any commercial products will be obtained.

Obviously, many further studies need to be carried out on these topics to advance these macrocyclic compounds from promising tools to clinical products. Scientists in many fields including clinical topics, chemistry, biology, biochemistry and materials science will be required. In the field of sensors, some of these areas are much less critical; for example, if the macrocycle is used in the determination of cancer in blood samples taken from the patient, then toxicology will not be an issue. However, for drug-like applications or use as drug-carrying hosts, we must fully understand the metabolism, the pharmacokinetics of the different systems, their biocompatibility as well as their breakdown, toxicology and excretion. Different methods of formulation need to be examined before these systems move from the laboratory to the clinic. However, although many of these studies are still in their early stages, they are showing great promise in the fields detailed within this review. The future could see these families of macrocycles being widely utilised in cancer detection, either directly or by detection of marker compounds, prevention (by detection or removal of carcinogens) and treatment, either as anti-cancer agents themselves or as carriers for chemotherapy drugs. The versatility of size, conformation and functionalisation of these macrocycles will enable them to perform with maximal efficacy and minimal toxicity.

## Figures and Tables

**Table 1:** Structural and physical parameters of cucurbiturils [5].

Cucurbituril	Cavity Width (nm)	Portal Width (nm)	Cavity Volume (nm^3^)
CB5	0.44	0.24	0.082
CB6	0.58	0.39	0.164
CB7	0.73	0.54	0.279
CB8	0.88	0.69	0.479
CB10	1.13–1.24	0.95–1.06	0.870

## Data Availability

Not applicable.
